# Nonlinear myocardial perfusion imaging with motion corrected reconstruction: validation via quantitative flow mapping

**DOI:** 10.1186/1532-429X-18-S1-O8

**Published:** 2016-01-27

**Authors:** Hui Xue, Michael S Hansen, Sonia Nielles-Vallespin, Andrew E Arai, Peter Kellman

**Affiliations:** grid.279885.90000000122934638National Heart, Lung, and Blood Institute, National Institutes of Health, Bethesda, MD USA

## Background

Myocardial perfusion imaging typically uses saturation recovery to generate T1 contrast during the Gd passage. Imaging protocols lead to a tradeoff between spatial/temporal resolution, myocardial coverage, and SNR. To improve resolution while maintaining the image quality we propose a nonlinear iterative reconstruction. This method explicitly integrates respiratory motion correction into the reconstruction to permit spatio-temporal regularization in the presence of motion. Unlike most k-t methods, the motion corrected images are directly output. In this way, complete free-breathing acquisition is achieved. Nonlinear iterative reconstruction is difficult to characterize since implicit filtering resulting from regularization is signal dependent. We propose to validate the proposed method by comparing quantitative myocardial blood flow (MBF) against linear reconstruction.

## Methods

In this study, the SPIRiT scheme [1] is extended to incorporate a motion correction operator which includes a forward and backward deformation. The unknowns are the motion corrected multi-channel complex images. A wavelet based L1-norm spatio-temporal regularization term is added, together with data fidelity and parallel imaging terms, to enforce signal consistency across different heart beats. Since motion fields are incorporated, the regularization can be more effective in suppressing random noise and aliasing as the tissue remains stationary. The regularization strength was experimentally selected to preserve dynamic changes of perfusion signal. All patients were approved by local IRB and written consent was collected. Imaging experiments were performed on a 3T clinical MRI system (MAGNETOM Skyra, Siemens). The administrated Gd dose was 0.075 mmol/kg for FLASH and 0.05 mmol/kg for SSFP. The proposed algorithm was implemented in C++ using the Gadgetron framework [2] and integrated inline on the scanner. A fully integrated Gadgetron Cloud based reconstruction [3] was used to further parallelize the computation.

## Results

**Figure 1:**
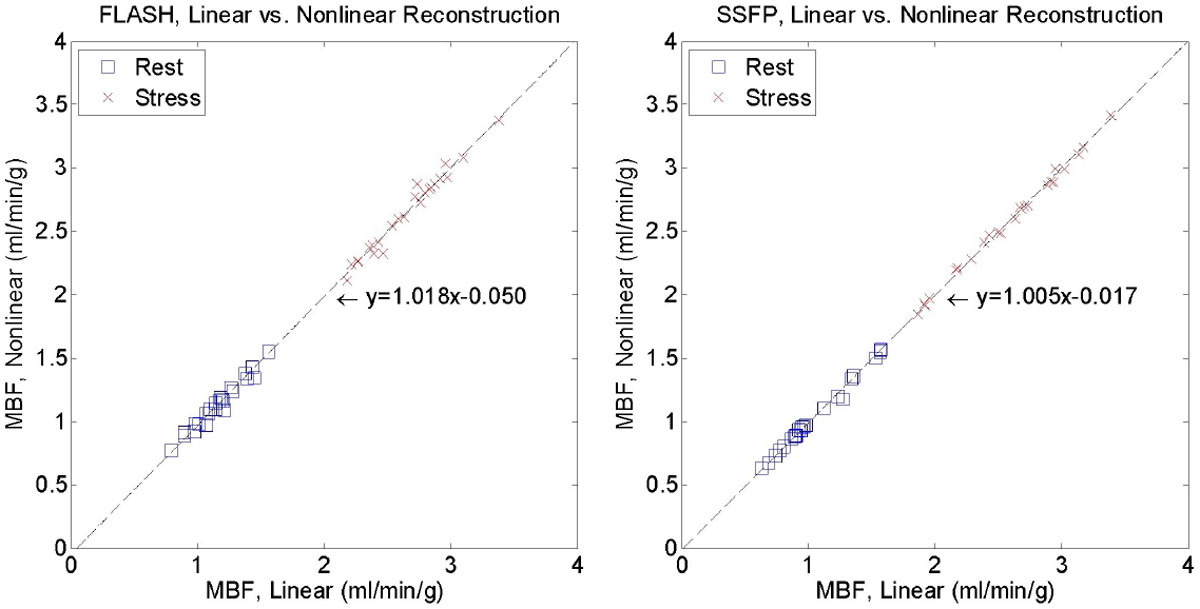
**N = 16 patients were scanned with FLASH (N = 8) and SSFP (N = 8) protocols which had moderate resolution: SR prepared FLASH/SSFP, 14°/50° flip angle, FOV 360 × 270 mm**^**2**^**, 8 mm slice thickness, 3 SAX slices, interleaved parallel acceleration R = 3, acquired matrix 192 × 111, ¾ partial Fourier, single shot imaging duration 53/67 ms**. Pixel-wise MBF maps were computed inline for linear and nonlinear reconstruction using a L1 model free deconvolution method. A ROI was drawn in the myocardium for every SAX slice. The mean MBF values for all FLASH rest/stress cases are 1.16 ± 0.19/2.65 ± 0.31 (linear) and 1.13 ± 0.19/2.65 ± 0.32 (nonlinear). For SSFP, mean MBF are 1.06 ± 0.30/2.58 ± 0.43 (linear) and 1.05 ± 0.29/2.58 ± 0.43 (nonlinear). No significant differences were found between linear and nonlinear flow values.

**Figure 2:**
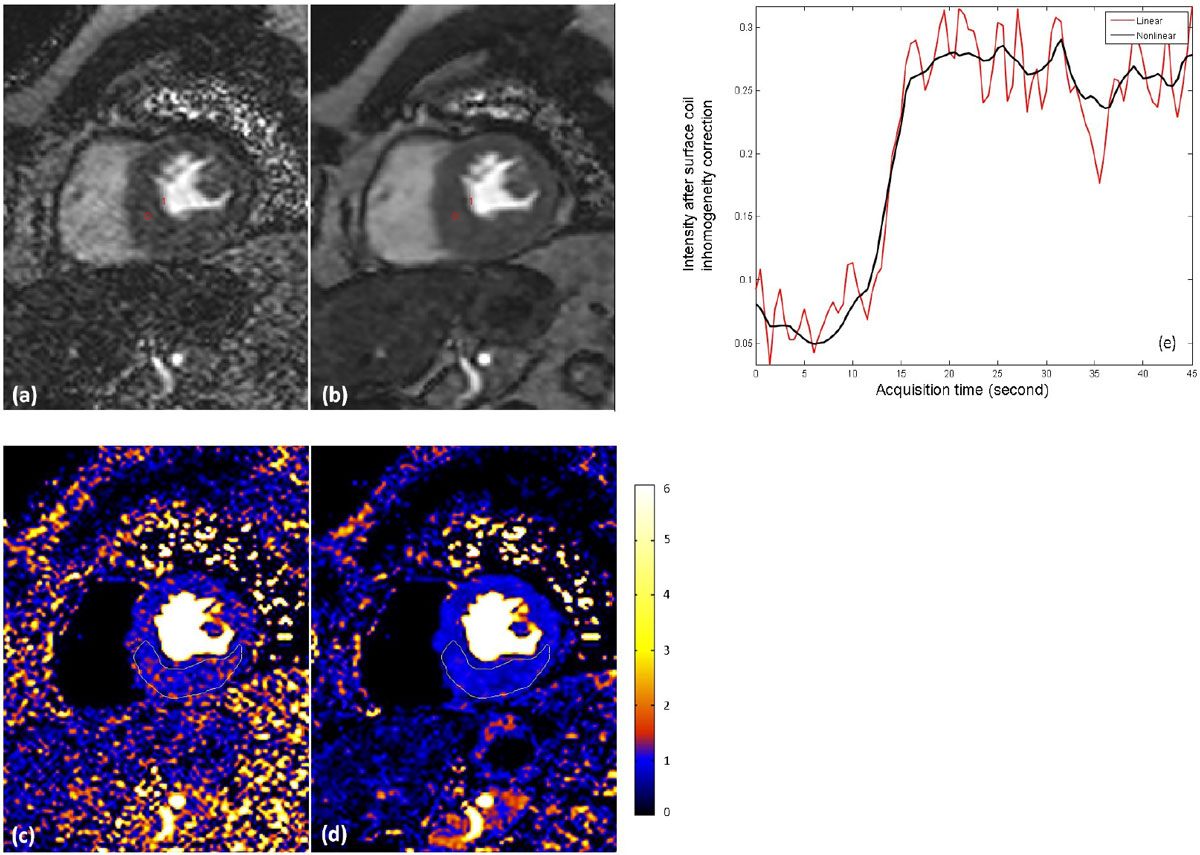
**In addition to the R = 3 protocols, 2 FLASH protocols with higher acceleration (R = 4) were tested: increased temporal resolution to 40 ms duration and increased spatial resolution to 256 × 144 matrix size**. An example of R = 4 256 × 144 rest perfusion FLASH study is shown here. (a) and (b) are surface coil inhomogeneity corrected perfusion images for GRAPPA and nonlinear method. The time intensity curves for a pixel selected in myocardium are plots in (e). The pixel-wise MBF maps are shown in (e) and (d). Due to the very poor SNR in the linear reconstruction, its MBF map is degraded, while the nonlinear MBF map gives much better quality. For a ROI drawn in the myocardium, the MBFs (ml/min/g) are 0.97 ± 0.33 (linear) and O.85 ± 0.16 (nonlinear), demonstrating the reduction of variability of MBF estimation.

The reconstruction time was ~400 s/150 s for three slices using single node and Gadgetron cloud version. For the standard protocol (R = 3), there were no significant differences in MBF (Fig. 1) between linear and non-linear reconstructions (t-test, p value, FLASH, rest/stress: 0.588/0.972; SSFP: 0.850/0.991). The high spatial resolution protocol with higher acceleration acquisition (R = 4) (Fig. 2) shows the reduction in noise for both images and flow maps. The time intensity curves preserve the dynamic characteristic with improved SNR using the proposed method.

## Conclusions

We propose a nonlinear iterative reconstruction with integrated respiratory motion correction. This method improves image quality and reduces variation in estimates of myocardial blood flow without introducing bias in MBF. With this technique, higher spatial and temporal resolution can be achieved.
